# Spike-Based Approximate Backpropagation Algorithm of Brain-Inspired Deep SNN for Sonar Target Classification

**DOI:** 10.1155/2022/1633946

**Published:** 2022-10-20

**Authors:** Yang Liu, Meng Tian, Ruijia Liu, Kejing Cao, Ruiyi Wang, Yadi Wang, Wei Zhao, Yi Zhou

**Affiliations:** ^1^Henan Province Engineering Research Center of Spatial Information Processing, Kaifeng 475004, China; ^2^College of Computer and Information Engineering, Henan University, Kaifeng 475004, China; ^3^Shenzhen Research Institute, Henan University, Shenzhen 518000, China; ^4^College of Software, Henan University, Kaifeng 475004, China; ^5^Henan Key Laboratory of Big Data Analysis and Processing, Kaifeng 475004, China; ^6^Miami College, Henan University, Kaifeng 475004, China; ^7^College of Artificial Intelligence, Henan University, Zhengzhou 450046, China

## Abstract

With the development of neuromorphic computing, more and more attention has been paid to a brain-inspired spiking neural network (SNN) because of its ultralow energy consumption and high-performance spatiotemporal information processing. Due to the discontinuity of the spiking neuronal activation function, it is still a difficult problem to train brain-inspired deep SNN directly, so SNN has not yet shown performance comparable to that of an artificial neural network. For this reason, the spike-based approximate backpropagation (SABP) algorithm and a general brain-inspired SNN framework are proposed in this paper. The combination of the two can be used for end-to-end direct training of brain-inspired deep SNN. Experiments show that compared with other spike-based methods of directly training SNN, the classification accuracy of this method is close to the best results on MNIST and CIFAR-10 datasets and achieves the best classification accuracy on sonar image target classification (SITC) of small sample datasets. Further analysis shows that compared with artificial neural networks, our brain-inspired SNN has great advantages in computational complexity and energy consumption in sonar target classification.

## 1. Introduction

Neural computing is the main driving force of the current development of artificial intelligence. In recent years, with the development of various deep learning technologies [1–5], artificial neural networks (ANNs) have been widely applied in many fields and achieved remarkable results (such as target detection, speech recognition, and character recognition). However, due to a large number of parameters, the massive training samples, and the huge energy consumption required by ANN training and deployment, ANN is still difficult to be applied to edge smart devices (such as smart watches, smart detectors, and other unmanned autonomous systems). High energy consumption and high-performance computing requirements have become the main bottlenecks for the continued development of neural networks for sonar target classification in unmanned underwater vehicles.

The brain-inspired spiking neural network (SNN) is usually sparse, and the calculation is driven by events. The high multiplication calculation costs of ANN can be avoided through discrete binary spike signals, showing ultralow power consumption [6]. Coordinate neuromorphic hardware [7–11] shows the prospect of realizing low-power artificial intelligence, which is expected to break through the current bottleneck of neural computing. The spiking neuron is inspired by biological neurons that efficiently process discrete spatiotemporal spikes, and the main neuronal models include the leaky integrate-and-fire (LIF) model [12], the Izhikevich model [13], and the Hodgkin–Huxley model [14]. LIF neurons greatly simplify the process of the action potential of biological neurons. The membrane potential integrates the input current over time, and when the membrane potential exceeds the threshold, the neuron will fire a spike. At present, there have been several hardware circuits [15] based on the LIF neuron model. Due to the discontinuity and nondifferentiability of spiking neuronal activation function, the traditional ANN backpropagation (BP) training algorithm based on gradient descent cannot be directly applied to brain-inspired SNN. The training of the brain-inspired SNN is still a great challenge. At present, the main work can be divided into two categories: the ANN-SNN conversion method and the brain-inspired SNN direct training method.

In the ANN-SNN conversion method, ANN is trained and then converted into SNN by a specific means to replace the training of SNN. Sengupta et al. [16] achieved 91.55% accuracy in the CIFAR-10 dataset with a loss of 0.15% conversion accuracy. After ANN-SNN conversion, Rathi et al. [17] fine-tuned the model for training and achieved 92.22% accuracy in the CIFAR-10 dataset and reduced the inference time. Stockl and Maass proposed a method for few spike conversion (FS-Conversion) [18], achieving 92.42% accuracy on the CIFAR-10 dataset. Because the state-of-the-art (SOTA) training methods of ANN are used, many current ANN-SNN conversion methods obtain SNN SOTA classification performance. However, the ANN-SNN conversion method usually imposes constraints on the original ANN, which will lead to a decline in performance. Moreover, most of the ANN-SNN conversion methods require hundreds to thousands of time steps to complete one inference, resulting in additional time delays and energy consumption contrary to the goal.

Direct training methods for SNN mainly include unsupervised learning and supervised learning. Unsupervised learning generally only involves local signals of synapses, such as the spike-timing-dependent plasticity (STDP) algorithm. Diehl and Cook [19] achieved a classification accuracy of 95% on the MNIST dataset using the STDP trained network. Kheradpisheh et al. [20] used STDP and the support vector machine (SVM) to achieve a classification accuracy of 98.4% on the MNIST dataset, but the accuracy was still far behind that of ANN. In order to narrow the gap, researchers put forward the spike-based BP rule. Jin et al. proposed the HM2-BP (hybrid macro/microlevel BP) [21] algorithm, and the error BP in SNNs was deconstructed into two processes: the change of postsynaptic potential caused by a single spike at the microlevel and the loss function defined by frequency coding at the macrolevel. Based on the approximate derivative of the spiking neuronal activation function, Wu et al. proposed a spatio-temporal backpropagation (STBP) [22] algorithm combining the spatial domain with the time domain in SNNs. Wu et al. also proposed a neural normalization technique (NeuNorm) [23], which achieved good results when combined with the STBP algorithm on CIFAR-10 datasets. Lee et al. [24] processed the derivative of LIF neurons as the approximate derivative of IF neurons and calculated the corresponding leak correction compensation. The authors in [25] proposed an SNN training algorithm that is capable of learning not only the synaptic weights but also the membrane time constants of SNNs. Literature [26] proposed a neuromorphic global-local synergic learning model. Compared with the single learning method, this method has much higher performance in few-shot learning. In addition, the authors in [27] proposed an SNN learning algorithm via proxy, which requires shorter simulation time than the converted SNNs. Previous SNN BP algorithms are more complex, but ANN BP algorithms are simple and effective.

The main contributions of our work are as follows: First, we propose the spike-based approximate backpropagation (SABP) algorithm for SNN training, whose approximate derivative of the spike neuronal activation function is simple and efficient. In addition, we have built general deep SNNs, which can adopt the popular architectures such as VGG [2] and ResNet [5] to build deep SNNs technologies and can also use SNN-based dropout to increase its generalization ability to alleviate the overfitting phenomenon in the learning process. We will then demonstrate the effectiveness of our work on MNIST, CIFAR-10, and sonar image target classification (SITC) datasets. To the best of our knowledge, the classification accuracy of our method is close to the best results on MNIST and CIFAR-10 datasets and achieves the best classification accuracy on the SITC dataset. Finally, we further analyze the advantages of this method compared with ANN in terms of computational complexity and energy consumption.

The structure of the paper is as follows: In Section 2, we will introduce our brain-inspired deep spiking neural network architecture and spike-based approximate backpropagation algorithm. In Section 3, the experiment is described and the experimental results are analyzed to demonstrate the effectiveness of the proposed method on MNIST, CIFAR-10, and SITC datasets. In Section 4, we discuss the relevant methods in recent years and compare them with ours. Finally, the thesis is summarized in Section 5.

## 2. Materials and Methods

### 2.1. Brain-Inspired Deep Spiking Neural Network Architecture

**Figure 1:**
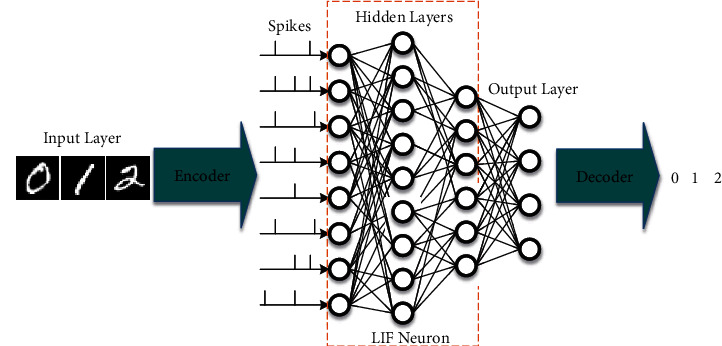
Overall architecture of brain-inspired SNNs. The encoder converts the input images into spikes, the hidden layers consist of LIF neurons, which can be any network structure (such as LeNet, VGG, and ResNet), and the decoder converts the output spikes into the corresponding classification results.

As shown in Figure 1, a deep SNN has been defined, including the input layer, encoder, hidden layer, output layer, and decoder. It is a feed-forward neural network, and the weight of each layer of neuron synapse will be updated according to the SNN BP algorithm described in Section 2.2. In this section, the structure and function of the encoder, the hidden layers, and the decoder will be mainly illustrated.

#### 2.1.1. Encoder

**Figure 2:**
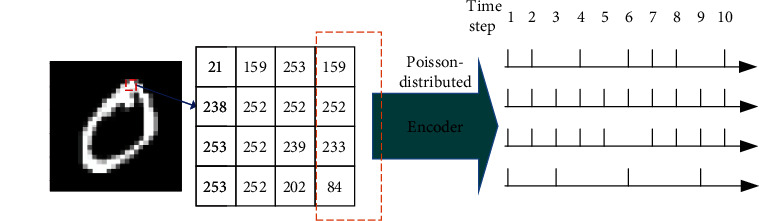
A simple example of the coding process. A 4 × 4 pixel image block is selected from the image, and the last column of the image block is transformed into the spike trains with a time step of 10, conforming to the Poisson distribution according to the pixel intensity.

As shown in Figure 2, in the encoder, the input image is two-dimensional static image data, the pixel image is encoded as a Poisson distributed spike train with a certain time step, and the probability of spike generation is proportional to the pixel intensity.

#### 2.1.2. Hidden Layers

**Figure 3:**
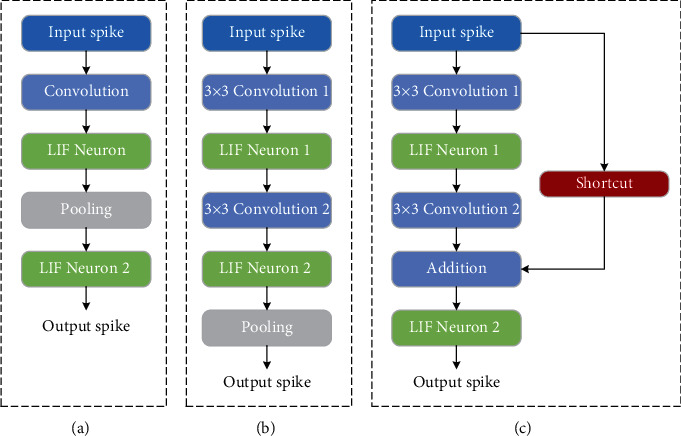
The basic architecture of the brain-inspired SNN blocks. (a) Spiking general structure. (b) Spiking VGG block. (c) Spiking ResNet block.

As shown in Figure 3, the hidden layers are composed of convolution layers, pooling layers, and fully connected (FC) layers. The features of spikes are extracted by the convolution layer and pooling layer, and then the one-dimensional vectors are generated in the FC layers and input to the decoder. The hidden layers are composed of neurons based on the LIF model. The leaking and firing processes of synapses in the convolutional layer and the pooling layer will be discussed in detail in Section 2.2.

#### 2.1.3. Decoder

**Figure 4:**
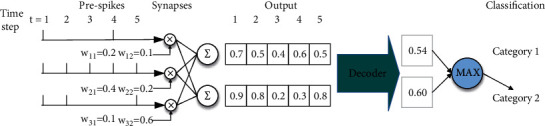
A simple example of the decoding process. In each time step, the value of the output layer is the result of multiplying the input spike train of the preneuron by the synaptic weight of the output layer. In the decoder, the values of the output layer in all time steps are accumulated and divided by the total time steps. Finally, the classification results are predicted by comparing the values of neurons in the decoder.

As shown in Figure 4, the decoder accumulates the one-dimensional vector features extracted from the output layer for final classification. This accumulation is the sum of the output spike of each time step multiplied by the weight of the corresponding output layer. The number of neurons in this layer is the same as the number of categories to be classified.

### 2.2. Spike-Based Approximate Backpropagation Algorithm

#### 2.2.1. Neuron Model

**Figure 5:**
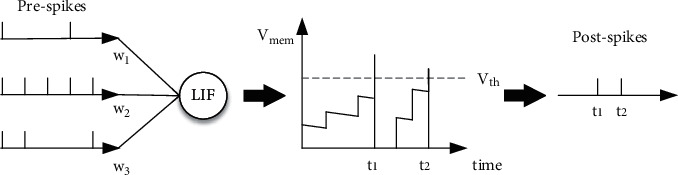
The process of leaky, integrate, and fire characteristics of LIF neurons. After integrating the current of the preneuron, the membrane potential of the postneuron begins to accumulate and decreases exponentially with time. Until the accumulation of the membrane potential exceeds the firing threshold, the LIF neuron fires a spike backward and resets the membrane potential.

As shown in Figure 5, the leakyintegrate-–and-fire (LIF) neuron model greatly simplifies the action potential process and retains the three key characteristics (leaky, integrate, and fire) of the neuron membrane potential, and its formula can be expressed as follows:

(1) $$\begin{array}{c} \tau_{m}\frac{dV_{mem}}{dt}=V_{rest}-V_{mem}+R_{m}I\left(t\right)\text{,} \end{array}$$

where *τ*_*m*_ is the time constant of membrane potential decays, *V*_*mem*_ is the postneuron membrane potential, *V*_*rest*_ is the resting potential, *R*_*m*_ is the impedance of the membrane, and *I*(*t*) is the input current.

#### 2.2.2. Spike Forward Propagation

**Figure 6:**
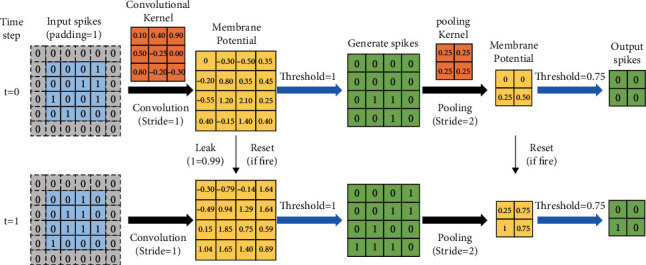
Illustration of a simplified operational example of the convolutional layer and the average pooling layer over two time steps. At each time step, when the membrane potential exceeds the firing threshold of the neuron, the postneuron generates an output spike and resets. If no spike is generated, the membrane potential will leak over time. In the average pooling layer, the membrane potential will not leak over time.

In forward propagation of spikes, the pixel value of the image is converted into a Poisson distributed spike train and transmitted to the network, and the input spike is multiplied by the synaptic weight to generate an input current. The input current accumulates in the membrane potential of the postneuron. When the membrane potential exceeds the firing threshold of the neuron, the postneuron generates an output spike and resets. If no spike is generated, the membrane potential decays (the membrane potential of the pooling layer neuron does not decay) exponentially over time. As shown in Figure 6, the neurons in each layer of the hidden layer will carry out this process in turn according to the input current received by the previous layer. As time goes by, the weighted sum of the spikes of the neurons can be formulated as follows:

(2) $$\begin{array}{c} net_{j}^{l+1}\left(t\right)=\overset{n^{l}}{\underset{i=1}{\sum }}w_{ij}^{l}\times x_{i}^{l}\left(t\right)\text{,} \end{array}$$

where *net*_*j*_^*l*+1^(*t*) represents the total current inflow of the membrane potential accumulated in neurons *j* in the layer *l*+1 over time *t*, *n*^*l*^ represents the total number of neurons in the layer *l*, *w*_*ij*_^*l*^(*t*) represents the weight of the connection synapse from the neuron *i* in the layer *l* to the neuron *j* in the layer *l*+1, and *x*_*i*_^*l*^(*t*) is the sum of the spike events of neurons in the layer *l* over time *t*, which can be formulated as follows:

(3) $$\begin{array}{c} x_{i}^{l}\left(t\right)=\overset{t}{\underset{k=1}{\sum }}f_{i}^{l}\left(t-t_{k}\right)\text{,} \end{array}$$

where *f*_*i*_^*l*^(*t* − *t*_*k*_) represents the moment when the neuron in the layer *l* generates a spike at the time *t*_*k*_, which can be expressed as follows:

(4) $$\begin{array}{c} f_{i}^{l}\left(t-t_{k}\right)=\left\{\begin{array}{cc} 1\text{,} & \text{if }\text{fire},\\ 0\text{,} & \text{otherwise.} \end{array}\right. \end{array}$$

In time *t*, the sum of the spike trains generated by the neuron *j* in the layer *l*+1 can be formulated by *a*_*j*_^*l*+1^(*t*) as follows:

(5) $$\begin{array}{c} a_{j}^{l+1}\left(t\right)=\overset{t}{\underset{k=1}{\sum }}f_{j}^{l+1}\left(t-t_{k}\right)\text{.} \end{array}$$

It can be seen that the sum of the spike trains produced by the neuron depends on the total amount of input current received. The neurons of the output layer will not generate spikes; instead, the membrane potential of the output layer will accumulate in the decoder at each time step, and the membrane potential of the decoder will not decay with time. At the last time step, the decoder will divide the accumulated membrane potential by the total time steps *T* to calculate the final result, which can be expressed as follows:

(6) $$\begin{array}{c} result=\frac{V_{mem}}{T}\text{.} \end{array}$$

#### 2.2.3. Error Backpropagation and Weight Update

After the forward propagation of a spike, the loss function is the difference between the predicted output of the decoder and the value of the label. At the decoder, the partial derivative of the loss function is calculated and propagated back to each previous layer using the chain rule, and the weight of each layer is updated according to the corresponding partial derivative obtained. The loss function can be expressed as follows:

(7) $$\begin{array}{c} L=\frac{1}{2}\overset{n^{l}}{\underset{j=1}{\sum }}\left(prediction_{j}-label_{j}\right)2\text{,} \end{array}$$

where *L* represents the final loss and *n* represents the total number of neurons in the decoder. The leak characteristic of LIF neurons is taken as noise; then, the partial derivative of the difference with respect to the weight parameter of the hidden layer can be expressed by the following formula:

(8) $$\begin{array}{c} \frac{\partial L}{\partial w}=\frac{\partial L}{\partial a}\frac{\partial a}{\partial net}\frac{\partial net}{\partial w}\text{,} \end{array}$$

where *w* represents the corresponding weight to be updated, *net* represents the input current of the preneuron, and *a* represents the output current of the neuron after activation. Different from ANN, the activation function in SNN represents the relationship between the weighted summation of the preneuronal input and the postneuronal output over time. If the membrane potential does not exceed the threshold, the neuron output is 0, and if the membrane potential exceeds the threshold, the neuron output is 1. Similar to the ReLU function, here, we only differentiate positive values in the network. Since the activation function of SNNs is discontinuous and nondifferentiable, it is necessary to find an approximate derivative for *∂a*/*∂net*. If the membrane potential of the LIF neuron does not exceed the firing threshold, no spike will be generated, and the derivative of the neuron activation function is set to 0. If the neuronal membrane potential exceeds the threshold, a spike will be generated, and the membrane potential of the postneuron at a given time instant *t* can be expressed by the following formula:

(9) $$\begin{array}{c} V_{mem}\left(t\right)\approx \overset{n}{\underset{i=1}{\sum }}\left(w_{i}x_{i}\left(t\right)\right)-V_{th}a\left(t\right)\text{,} \end{array}$$

where *n* represents the number of preneurons, *x*_*i*_(*t*) represents the sum of the spikes of the pre-neuron *i* over time *t*, and *a*(*t*) represents the sum of the spikes after the activation of the postneuron over time *t*. Since *V*_*mem*_(*t*) does not reach the firing threshold, *V*_*mem*_(*t*) will be ignored, and then, the derivative of the activation function can be approximated as a linear function; it can be directly estimated as 1/*V*_*th*_ [24], and the formula is deduced as follows:

(10) $$\begin{array}{c} a\left(t\right)\approx \frac{1}{V_{th}}\overset{n}{\underset{i=1}{\sum }}\left(w_{i}x_{i}\left(t\right)\right)\text{.} \end{array}$$

According to formula (2), it can be obtained as follows:

(11) $$\begin{array}{c} a\left(t\right)\approx \frac{1}{V_{th}}net\left(t\right)\text{,} \end{array}$$

(12) $$\begin{array}{c} \frac{\partial a}{\partial net}=\frac{1}{Vth}\text{.} \end{array}$$

Then, the partial derivative of the difference with respect to the weight can be approximated as follows:

(13) $$\begin{array}{c} \frac{\partial a}{\partial w}=\frac{1}{Vth}\frac{\partial L}{\partial a}\frac{\partial net}{\partial w}\text{.} \end{array}$$

For simplicity, *V*_*th*_ is set to 1 in this paper so that the formula can be simplified as follows:

(14) $$\begin{array}{c} \frac{\partial L}{\partial w}=\frac{\partial L}{\partial a}\frac{\partial net}{\partial w}\text{.} \end{array}$$

#### 2.2.4. Dropout in the Spiking Neural Network

Dropout [3] is a very popular regularization technology for training ANN. In the training process, given a probability *p* that obeys the Bernoulli distribution, some neurons are randomly disconnected by *p* to avoid the occurrence of overfitting phenomenon.

In SNN, the use of dropout is slightly different from that of ANN. In the training process of ANN, data are divided into several batches, each iteration has only one forward propagation, and a number of connections between neurons and networks are disconnected randomly according to the probability *p*. However, in SNN, there will be multiple forward propagations in each iteration, which depends on the time step set. The error is back propagated, and the network parameters are updated only at the last time step. Therefore, the consistency of disconnection neurons in each time step must be guaranteed in an iteration. In ANN, the typical dropout probability *p* is set to 0.5. Since the activation of neurons is sparser in the forward propagation of SNN, the set of probability *p* in SNN is generally smaller than that of ANN. In our work, it is generally set to somewhere between 0.1 and 0.25.

## 3. Experiments and Results

### 3.1. Dataset Description

In order to measure the effectiveness of the SNN BP training algorithm in the brain-inspired SNN model, MNIST, CIFAR-10, and SITC datasets are selected for experiments. The MNIST handwritten dataset is composed of grayscale images with an image size of 28 × 28. There are 60,000 training samples and 10,000 test samples, including 10 digital categories of 0–9. CIFAR-10 is composed of color images with an image size of 32 × 32. There are 50,000 training samples and 10,000 test samples, including 10 categories of animals and vehicles. SITC is a small-sample sonar image dataset. The SITC dataset is based on 62 aircraft images and 385 shipwreck images publicly provided by the SeabedObjects-KLSG [28] dataset, and 289 seabed images and 18 drowning images are collected and combined into an experimental dataset for underwater target classification. All target images are directly cropped from the original sonar image. A total of 18 drowning images, 62 aircraft images, 289 seabed images, and 385 shipwreck images are included.

Table 1 lists the detailed information about the experiments' datasets. The training samples of the three datasets are used for deep SNN training, and then, the trained SNN is used to predict and classify the test samples of the dataset to obtain classification accuracy. There is no intersection between training samples and test samples.

### 3.2. Experimental Setup

A custom simulation the SNN framework is developed using the PyTorch deep learning framework, which is available in Python 3.6.12 and PyTorch 1.1.0. For training, as described in Subsection 2.2.3, synaptic weights are trained with a mini-batch approximate BP algorithm in an end-to-end manner. A stochastic gradient descent (SGD) optimization algorithm is used to optimize the training parameters, and each experiment has 150 training epochs.

### 3.3. Network Topologies

As described in Section 2.1, the hidden layer of SNN can contain the current popular network architecture. The appropriate SNN architecture is selected according to the complexity of the experimental dataset. For the convenience of comparison, a network architecture similar to the LeNet5 model is used on the MNIST and SITC datasets, which contain two sets of convolutional layer pooling layers and two FC layers. For the CIFAR-10 dataset, a deeper network architecture is chosen, with a structure similar to ResNet11. The hidden layer is composed of residual blocks, and there are 11 layers of trainable parameters. The detailed structure is shown in Table 2.

### 3.4. Encoding Scheme

For the MNIST dataset, the pixel value of the grayscale image is scaled between 0 and 1 and then transformed into a spike event stream with a certain number of time steps conforming to the Poisson distribution according to the pixel intensity. For the CIFA-10 dataset, we first preprocessed the color image by horizontal flipping, scaling the pixel value between −1 and 1 and then transformed it into a spike event stream with a certain number of time steps in line with the Poisson distribution according to the pixel intensity. For the SITC dataset, the pixel value of the gray image is scaled to between 0 and 1 and then transformed into a spike event stream with a certain number of time steps conforming to the Poisson distribution according to the pixel intensity.

In SNN, the spike event for each time step can only be 0 or 1, and the time steps provide additional time dimension information; they can be considered as the actual precision of neuronal activation. If the time steps are too less, SNN will not be able to get enough information for inference. If the time steps are too long, the randomness of SNN will be destroyed, and the additional inference time and energy consumption will be generated. In our experiments, the time steps of the relatively simple MNIST dataset are 50, the time steps of the more complex CIFAR-10 dataset are 100, and the time steps of the SITC dataset are 70.

### 3.5. Experimental Results

At present, MNIST and CIFAR-10 are mostly used to verify SNN inference ability. Tables 3–5, respectively, list the classification results of SNN on MNIST, CIFAR-10, and SITC datasets in recent years, and the results of our algorithm SABP are bold in thetable.

As shown in Tables 3–5, the accuracy of the SABP algorithm is 99.62%, 91.03%, and 91.11%, respectively. For MNIST and CIFAR-10 datasets, the SABP algorithm is very close to the highest accuracy of surrogate gradient [25] (Tables 3 and 4). For the SITC dataset of the small sample, after image enhancement by style transfer and weighted random sampling, the classification accuracy is significantly higher than that of surrogate gradient [25] (Table 5).

## 4. Discussion

We propose the general brain-inspired SNN framework and the SABP algorithm. The SABP algorithm derives the approximate derivative of an LIF neuron by the relationship between the input current of the LIF neuron and the output of the neuron after activation, and in this process, we treat the leak characteristics of LIF neurons as noise. In the weight update stage, we use the approximate derivative to realize the error backpropagation. The approximate derivative of the SABP algorithm is very concise because we believe that an SNN training algorithm must be concise and effective.

### 4.1. Comparison with Relevant Works

Unlike the HM2-BP algorithm proposed by Jin et al. [21] and the STBP algorithm by Wu et al. [22], our work is similar to that of the approximate derivative algorithm of IF neurons by Lee et al. [24], which considers only the activation of spikes to naturally train the deep brain-inspired SNN. In addition, in the process of training, the method by Lee et al. in each neuron is to save a complex leakage compensation value for error backpropagation. However, our LIF neuron activation function approximate derivative is very simple (active 1 and inactive 0) and without additional storage required, which saves the memory and improves the training speed. Therefore, the work in this paper can advance the spike-based BP algorithm for training deep SNN.

### 4.2. Calculation of Energy Consumption

Different from ANN, which requires multiply-accumulate computation (MAC) at each layer, the spikes of SNN are sparse and only have accumulate computation (AC) at each layer, which enables SNN to reduce computational complexity and save energy consumption. If both SNN and ANN use conventional hardware, this advantage will be lost, and even the computational complexity will be tens to hundreds of times that of ANN. Fortunately, special hardware is available to implement event-based operations, and SNN can take advantage of this mechanism to compare with ANN.

**Figure 7:**
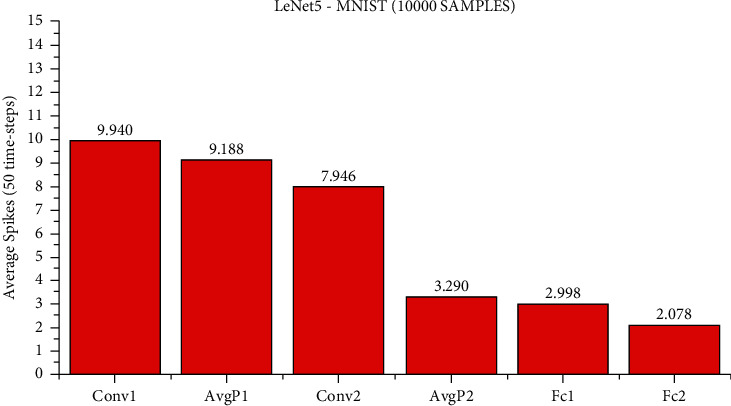
The average number of spike activations at each layer of LeNet5. On all test sets of MNIST samples, when using 50 time steps, the accuracy of this method is 99.62%, and the average number of spikes activated per layer under the LeNet5 network architecture is obtained.

**Figure 8:**
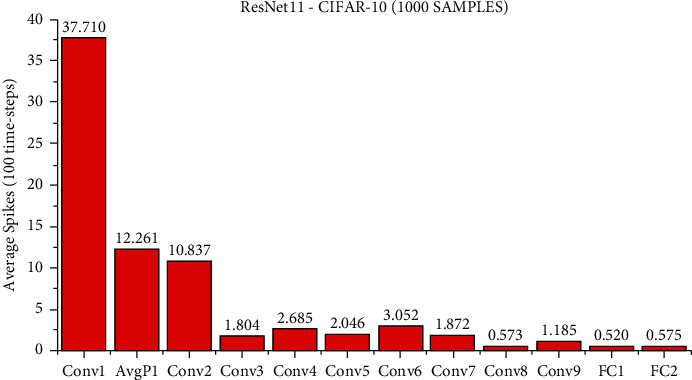
The average number of spike activations at each layer of ResNet11. When using 100 time steps, 1000 samples are randomly selected from the test samples of the CIFAR-10 dataset, and the accuracy of this method is 92.00%; the average number of spikes activated per layer under the ResNet11 network architecture is obtained.

**Figure 9:**
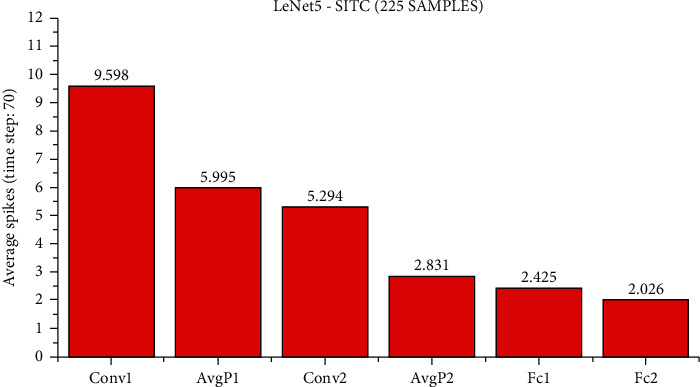
On all test sets of MNIST samples, when using 70 time steps, the accuracy of this method is 91.11%, and the average number of spikes activated per layer under the LeNet5 network architecture is obtained.

At present, although it is difficult for us to directly estimate the actual energy consumption of SNN and ANN, we can still make a rough estimate of the synaptic operands of each network layer to compare the computational complexity of SNN and ANN. Suppose an SNN has *l* layers, each layer has *n*_*l*_ synaptic connections, the total time steps are *T*_*s*_, and the average activation rate of synapses is *a*_*l*_, then the AC operand of an SNN is ∑_*l*_*n*_*l*_ × *a*_*l*_ × *T*_*s*_, and ∑_*l*_*n*_*l*_ × *a*_*l*_ is the MAC operand of an ANN of the same structure. The number of spike activations at each layer of LeNet5 and ResNet11 is shown in Figures 7–9.

We use the calculation method described above, under the LeNet5 architecture, the AC operands of SNN are about 7.4x of the MAC operand of ANN. Under the ResNet11 architecture, the AC operands of SNN are about 2.2x as many as the MAC operands of ANN. However, according to the work by Han et al. [35], the energy of MAC operation is usually one order of magnitude higher than that of AC operation. For example, in the 45 nm technology node, the energy consumed by 32-bit floating-point MAC operation is about 4.6PJ and that of AC operation is about 0.9PJ. The power consumption for 32-bit integer MAC operation is about 3.2 PJ, and for AC operation, it is about 0.1 PJ. It has been reported [36] that 32-bit floating-point computing can be replaced by fixed-point computing using integer MAC and AC units with almost no loss. According to the calculation method mentioned above, for the MNIST dataset, the calculation energy efficiency of SNN is about 4.32x that of ANN, for the CIFAR-10 dataset, the computational energy efficiency of SNN is about 14.55x that of ANN, and for the SITC dataset, the computational energy efficiency of SNN is about 6.2x that of ANN. It is worth noting that the average activation rate of neurons in each layer decreases with the deepening of the SNN network, and the activated neurons become more and more sparse. Although the actual energy consumption of SNN and ANN is not completely consistent with the synaptic operand and the actual energy consumption may be affected by other factors, the advantages of brain-inspired SNN in computational complexity and energy consumption can still be observed.

## 5. Conclusion

Aiming at the problem that CNN is difficult to deploy on edge smart devices due to high energy consumption, this paper proposes a general brain-inspired SNN architecture and a simple and effective SABP algorithm and constructs different network architectures according to the characteristics of different datasets. Experiments show that the method has a good performance on brain-inspired deep SNN. Energy consumption analysis proves the superiority of SNN in energy consumption, which is more suitable for deploying edge smart devices than CNN. So far, compared with other SNN training methods, the accuracy of our experiment is very close to the highest accuracy on MNIST and CIFAR-10 datasets, and the highest accuracy is achieved on small-sample SITC datasets. When applied to suitable neural mimicry hardware, our proposed approach can significantly reduce computational complexity and energy consumption for sonar target classification in unmanned underwater vehicles.

In the future, we will strive to reduce the time step required for SNN classification to reduce the delay and deploy the algorithm on an unmanned underwater vehicle equipped with neuromorphic hardware for further research.

## Figures and Tables

**Table 1:** Experiments datasets.

Dataset	Image	Train sets	Test sets	Category
MNIST	28 × 28 gray	60000	10000	10
CIFAR-10	32 × 32 × 3 color	50000	10000	10
SITC	Unfixed gray	529	225	4

**Table 2:** LeNet5 and ResNet11 network architecture.

LeNet5				ResNet11			
Layer	Kernel	Channel	Stride	Layer	Kernel	Channel	Stride
Conv	1 × 5 × 5	20	1	Conv	3 × 3 × 3	64	1
Avgpool	2 × 2	20	2	Avgpool	2 × 2	20	2
Conv	20 × 5 × 5	50	1	Conv	64 × 3 × 3	128	1
Avgpool	2 × 2	50	2	Conv	128 × 3 × 3	128	1
				Shortcut	64 × 1 × 1	128	1
				Conv	128 × 3 × 3	256	1
				Conv	256 × 3 × 3	256	2
				Shortcut	128 × 1 × 1	256	2
				Conv	256 × 3 × 3	512	1
				Conv	512 × 3 × 3	512	1
				Shortcut	256 × 1 × 1	512	1
				Conv	512 × 3 × 3	512	1
				Conv	512 × 3 × 3	512	2
				Shortcut	512 × 1 × 1	512	2
FC		200		FC		1024	
Output		10		Output		10	

**Table 3:** The classification accuracy of SNN on the MNIST dataset.

Author	Method	Accuracy (%)
Diehl and Cook [19]	STDP	95.00
Kheradpisheh et al. [20]	STDP	98.40
Hunsberger and Eliasmith [29]	Conversion	98.37
Diehl et al. [30]	Conversion	99.10
Rueckauer et al. [31]	Conversion	99.44
Lee et al. [32]	Spike-based BP	99.31
Jin et al. [21]	HM2-BP	99.49
Wu et al. [22]	Spike-based BP	99.42
Lee et al. [33]	STDP + spike-based BP	99.28
Lee et al. [24]	Spike-based BP	99.59
Fang et al. [25]	Surrogate gradient	99.72
Wu et al. [26]	Spike-based HP	99.50
**This work**	**SABP**	**99.62**

**Table 4:** The classification accuracy of SNN on the CIFAR-10 dataset.

Author		Method		Accuracy (%)
Hunsberger and Eliasmith [29]		Conversion		82.95
Esser et al. [34]		Conversion		89.32
Rueckauer et al. [31]		Conversion		88.82
Sengupta et al. [16]		Conversion		91.55
Rathi et al. [17]		Conversion + STDB		92.22
Stöckl and Maass [18]		FS-conversion		92.42
Wu et al. [23]		Spike-based BP		90.53
Lee et al. [24]		Spike-based BP		90.95
Fang et al. [25]		Surrogate gradient		93.50%
Saeed Kheradpisheh and Maryam [27]		Proxy		93.11%
Wu et al. [26]		Spike-based HP		91.08%
This work		SABP		91.03

**Table 5:** The classification accuracy of SNN on the SITC dataset.

Author		Method		Accuracy (%)
Fang et al. [25]		Surrogate gradient		86.67%
This work		SABP		91.11

## Data Availability

The datasets used to support the findings of this study are available at MNIST DATASET (http://yann.lecun.com/exdb/mnist/) CIFAR10 DATASET (https://tensorflow.google.cn/datasets/catalog/cifar10).
